# Chest CT features of COVID-19 pediatric patients presented with upper respiratory symptoms

**DOI:** 10.1186/s43055-021-00511-3

**Published:** 2021-05-31

**Authors:** Marwa Samy, Lamiaa M. R. Khalaf

**Affiliations:** 1 Radiology Department, Faculty of Medicine, Assiut University, Asyut, 71111 Egypt; 2 Radiology Department, South Egypt Cancer Institute - Assiut University, 8 El Mesak Street Branch of King Seti, Asyut, 71111 Egypt

**Keywords:** COVID-19, Pediatric, Chest CT, Respiratory

## Abstract

**Background:**

An outbreak of coronavirus disease 2019 (COVID-19) infection caused by a novel coronavirus began in December 2019 in Wuhan in central China that affect the children and adult and causes respiratory disease. This study aimed to highlight the CT features of pediatric patients with COVID-19 with upper respiratory symptoms.

**Results:**

The mean age of the 53 patients who enrolled in this study were 14.06 ± 4.12 years (range 1-18 years). Majority (75.5%) of them were adolescent**.** Running nose and fever were the most common symptoms. Forty-four (83%) patients had normal CT with no ground glass opacity (GGO) or consolidation, only 9 patients showed lung opacities; 5 cases showed consolidation**,** 2 cases with GGO, while consolidation with GGO was noted in another 2 patients**.** Among these 9 patient, six (66.7%) of them show unilateral lung opacities with peripheral distribution of these opacities in eight (88.9%) patients. The most frequently affected lobes were right and left lower lung lobes that were noted in 6 out of 9 patients with lung opacities (66.7%) in each lobe**.** Bronchitis was predominant in 23 (43.4%) cases.

**Conclusions:**

RT-PCR is a gold slandered test in the diagnosis of COVID-19 in children with upper respiratory tract symptoms as Chest CT cannot standalone as a diagnostic tool owing to high prevalence of normal chest CT in these patients. However, in case of lung affection, the presence of unilateral, peripheral consolidation with lower lobe predominance, in addition to bronchial wall thickening should be considered as a hallmark of chest CT in diagnosis of pediatric patients with COVID-19 with upper respiratory symptoms.

## Background

Multiple cases of an unexplained respiratory disease discovered in December 2019 in Wuhan, China. Laboratory analysis of respiratory samples revealed a novel coronavirus (SARS-CoV-2), that causes a coronavirus disease 2019 (COVID-19). The cases of COVID-19 were rapidly increased in China and globally [1]. The World Health Organization (WHO) declared COVID-19 as pandemic disease on March 11, 2020 [2].

Symptoms of coronaviruses are similar to other pneumonias, such as severe acute respiratory syndrome (SARS) and Middle East respiratory syndrome (MERS), all of them presented by sore throat, malaise, fever, cough, dyspnea, and parenchymal pulmonary changes on MSCT chest [3]. Reports of SARS in 2003 showed that all age groups were susceptible to infection, but children appeared to be less affected by the disease, with fewer and less severe cases [4]. The exact number of pediatrics worldwide affected by SARS is not clearly known due to incomplete age classification in the reported cases. It was illustrated that only about 5% of people affected were younger than 18 years old, with no reported child deaths [5].

Radiological imaging, clinical, and epidemiological studies of COVID-19 recently have emerged; unfortunately, most of them have focused on adult cases, with a little reporting findings in children. In this study, we conducted a retrospective analysis of the computed tomographic (CT) features of COVID-19 in pediatric patients in order to assess the value of chest CT as a diagnostic tool for COVID-19 infection in children presented with upper respiratory tract symptoms and to familiarize the radiologists with possible CT patterns of COVID-19 in children.

## Methods

### Patients selections

This retrospective study was approved by our Institutional Ethics Committee. From April 2020 to July 2020, 53 consecutive pediatric patients who confirmed laboratory to be positive for COVID-19 virus and presented with upper respiratory symptoms were enrolled in this study and underwent chest CT. All patients were confirmed to have COVID-19 nucleic acid on the basis of positive findings for respiratory secretions tested using real-time reverse-transcription–polymerase chain reaction (RT-PCR) obtained by nasopharyngeal or oropharyngeal swab. Patients with excessive motion that causes artifacts on CT chest and those with lower respiratory symptoms (cough and dyspnea) were excluded from this study.

The patients were divided into four groups: Toddler group from 1 to 3 years, preschool group from 3 to 6 years, school group from 6 to 12 years, and the adolescent group patient from 12 to 18 years [6]. Clinical symptoms of upper respiratory tract infection such as fever, sore throat, running nose, and headache were analyzed for all children.

### CT acquisition

Chest CT examinations were obtained using multi-detectors CT (MDCT) scanners (16-slice CT, bright speed, GE Healthcare Technologies) without intravenous contrast material. The scanning range was set from the level of thoracic inlet to the lowest costophrenic angle. The imaging parameters were as follows: Tube voltage, 100-120 KV; mA, automatic exposure control; pitch, 1.375; collimation 2 mm; reconstruction slice thickness, 0.5mm; interslice gap, 0 mm; and the tube current exposure time, 200-300 mAs.

During chest CT examination, 10% chloral hydrate (5-10 mg/kg that is administrated orally 30 min before the scan) was given to children who are unable to cooperate or those under 3 years of age to ensure the calm breathing during the scan.

### CT analysis

The CT scans were independently reviewed by two radiologists (LMR and MS, with 7 and 9 years thoracic radiology experience respectively), who were blinded to the clinical information and the final decision was taken with consensus. The following CT features were analyzed in each patient: (1) Lesion density: (a) pure ground-glass opacity (GGO) which was defined as increased lung attenuation with preservation of the vascular margins and bronchial wall, (b) pure consolidation (defined as opacification with obscuration of underlying vascular margins and airway wall), (c) mixed GGO and consolidation; (2) Laterality of the lung opacities: (a) unilateral, (b) bilateral parenchymal lung affection with opacities; (3) Distribution of the lung opacities: (a) peripheral (involving most the outer 1/3 of the lungs), (b) central (involving most the inner 2/3 of the lungs), or (c) both central and peripheral (no definite clear predominance); (4) Affected lobes; (5) Number of affected lobes; (6) Other findings were also reported such as (a) vascular thickening, (b) bronchial wall thickening, and (c) pleural thickening (thickness more than 3 mm).

### Statistical analysis

Statistical analysis was performed with software (SPSS, version 22.0, IBM). Data were recorded using spreadsheet software (Excel 2013, Microsoft). Quantitative variables were expressed as mean (standard deviation, SD) or median (range) values and nominal data were expressed in the form of frequency (percentage). The frequency of CT signs was expressed as the number (percentage) of occurrence.

## Results

**Fig. 1:**
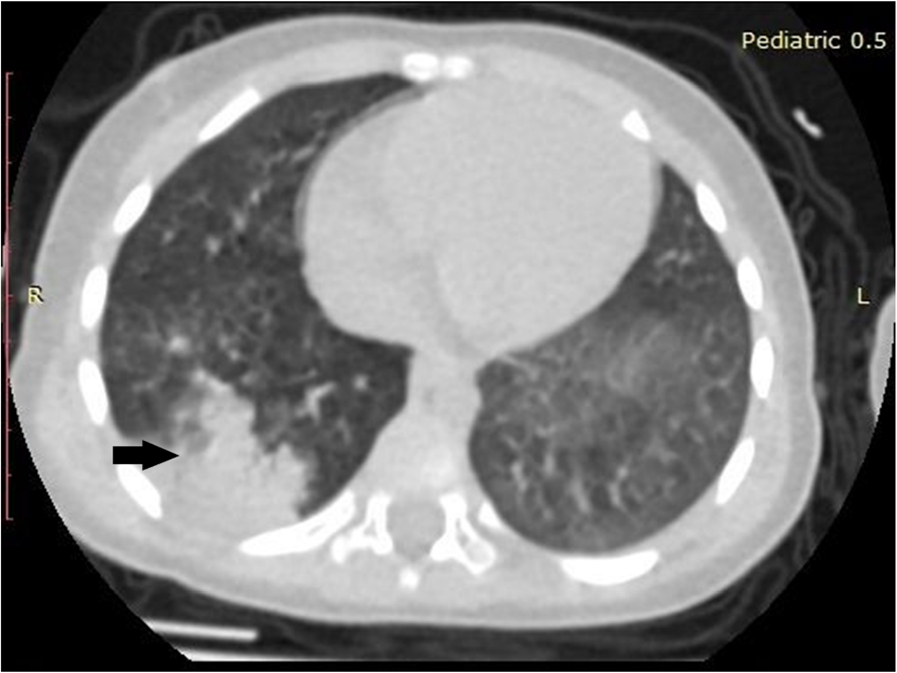
Axial non-contrast enhanced CT chest of 3-year-old boy, 2 days after the onset of fever and sore throat, there is a unilateral peripherally based consolidation in the lateral and posterior segments of the right lower lung lobe (black arrow)

**Fig. 2:**
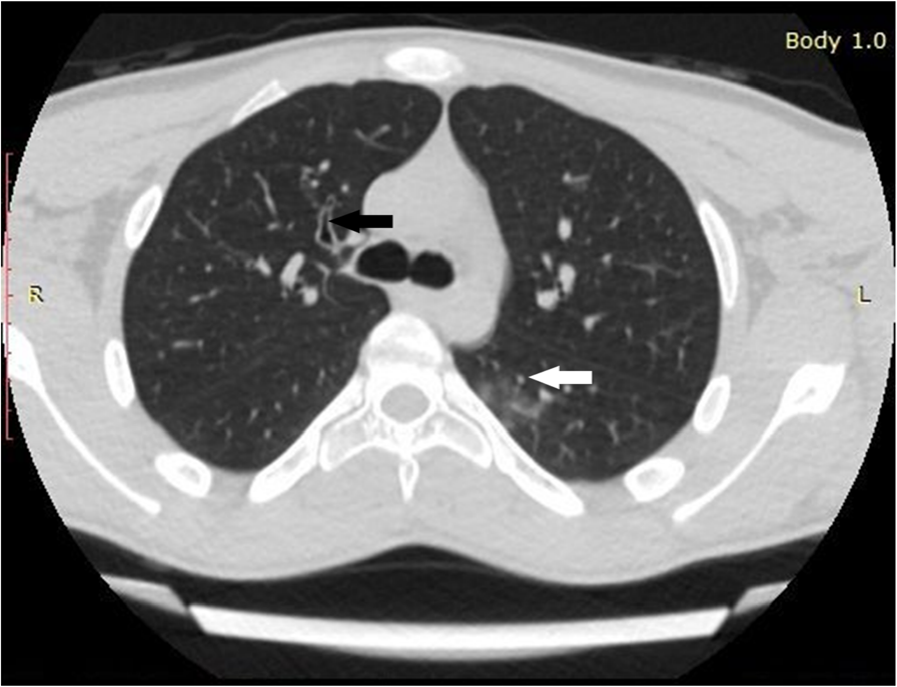
Axial non-contrast enhanced chest CT of 14-year-old boy, 1 day after the onset of fever and running nose, there is unilateral GGO in the superior segment of the LT lower lung lobe (white arrow) and bronchial wall thickening in the anterior segment of the RT upper bronchus (black arrow)

**Fig. 3:**
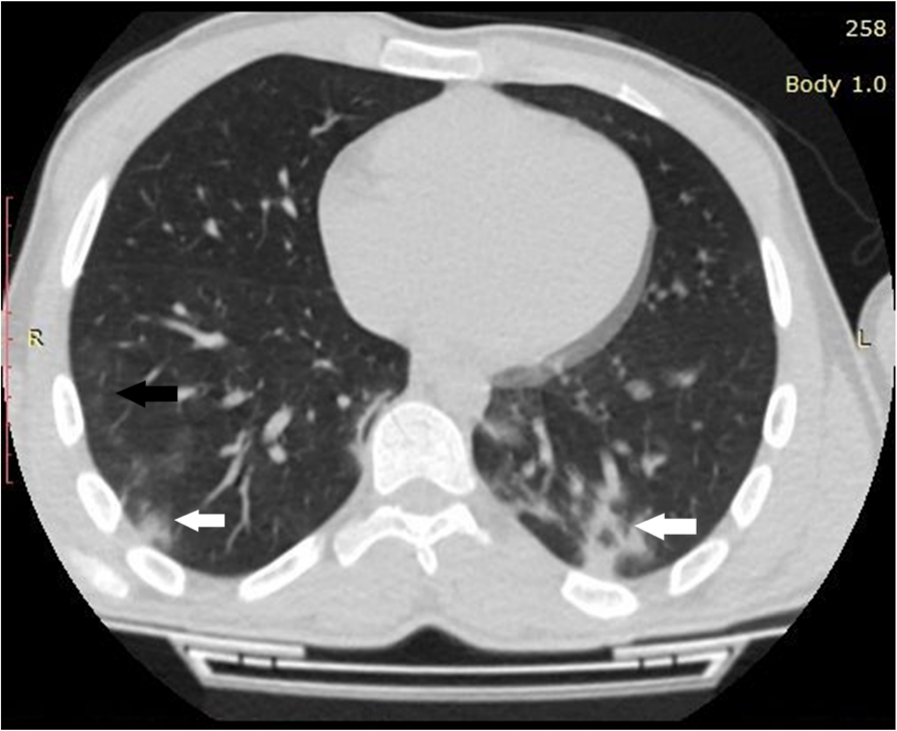
Axial non-contrast enhanced CT chest of 16-year-old girl, 2 days after the onset of fever, there are patches of ground glass opacities (black arrow) with consolidation (white arrow) distributed peripherally in the posterior segments of both lower lung lobes

Mean age of the enrolled 53 patients was 14.06 ± 4.12 years (range 1-18 years). Twenty-seven (50.9%) patients were females and 26 (49.1%) patients were males. Majority (75.5%) of those patients were adolescent (Table 1). Running nose and fever were the most common symptoms in the examined patients (Table 2). Forty-four (83%) patients had normal CT with no GGO or consolidation, only 9 patients showed lung opacities; 5 cases showed consolidation (Fig. 1), 2 cases with GGO (Fig. 2), and consolidation with GGO was noted in 2 patients (Fig. 3). Among these 9 patients, six (66.7%) of them show unilateral lung opacities (Figs. 1 and 2) with peripheral distribution of these opacities in eight (88.9%) patients (Figs. 1, 2, 3). The most frequently affected lobes were right and left lower lung lobes that were noted in 6 out of 9 patients with lung opacities (66.7%) in each lobe (Table 3). Bronchitis was predominant in 23 (43.4%) cases (Fig. 2).

**Table 1:** Age group distribution among the examined patients

Age group	N = 53
Adolescent	40 (75.5%)
School	9 (17%)
Toddler	2 (3.8%)
Preschool	2 (3.8%)

Data expressed as frequency (percentage)

**Table 2:** Presenting clinical symptoms of the studied patients

Clinical symptoms	Number of patients
Running nose	35 (66%)
Fever	34 (64.2%)
Sore throat	10 (18.9%)
Headache	1 (1.9%)

Data expressed as frequency (percentage)

**Table 3:** Chest CT findings of the enrolled patients

Lesion density	
Normal	44 (83%)
Consolidation	5 (9.4%)
GGO	2 (3.8%)
GGO and consolidation	2 (3.8%)
**Laterality**	
Normal	44 (88.7%)
Unilateral	6 (11.3%)
Bilateral	3 (5.7%)
**Lesion distribution**	
None	44 (83%)
Peripheral	8 (15.1%)
Peripheral and central	1 (1.9%)
**Affected lobes**	
Right lower lobe	6 (11.3%)
Left lower lobe	6 (11.3%)
Right upper lobe	2 (3.8%)
Left upper lobe	2 (3.8%)
Right middle lobe	1 (1.9%)
**Other CT findings**	
Bronchitis	23 (43.4%)
Vascular thickening	3 (5.7%)
Pleural thickening	1 (1.9%)

*GGO* ground-glass opacity

Data expressed as frequency (percentage)

## Discussion

Children who had COVID-19 infections presented with upper respiratory symptoms were difficult to diagnose. PCR is described as the gold standard for COVID-19, but it has falsely negative results due to improper sampling that requires repeated samples **[**7]. It is unknown whether CT scanning has additional value as diagnostic tool to rule out COVID-19 in children presented with upper respiratory symptoms [8]. It would require very convincing evidence to justify the introduction of ionizing radiation to rule out COVID-19 infection in children.

Most of our examined pediatric patients were in the adolescent group. Running nose and fever were the most common clinical symptoms in the examined patients, these symptoms are in concordance with previous literature that was done by Chen et al. [8] who reported that running nose and fever were the common symptom in children with COVID-19. Also, the clinical severity classification of the COVID-19 guidelines in China stated that pediatric patients having milder symptoms than adult [9].

Regarding the CT features of the enrolled cases, most of them (83%) had normal chest CT, these results were largely consistent with multiple previous studies [10–12], which reported that most of the pediatric patients had normal chest CT, and also had less severity than adult on imaging. Also, the study done by Steinberger et al. [13] in 30 pediatric patients as in our study stated that 77% of their children patients had negative CT findings. From these results, we conclude that the radiologist should not be fooled by the normal CT chest to exclude the COVID-19, subsequently CT chest cannot standalone to rule out COVID-19 infection in children, so PCR testing is essential for making the diagnosis of COVID-19 in children presented with upper respiratory tract symptoms as early isolation of these patients to reduce human to human transmission is necessary. Moreover, the Society of Thoracic Radiology and the American College of Radiology do not support the use of chest CT for routine screening of COVID-19 in children, keep in mind the hazards and potential risks of ionizing radiation [14].

It is noteworthy that, the most commonly lung parenchymal opacity in our pediatric patients with positive CT findings was lung consolidation which was observed in more than half (55.6%) of the cases with lung opacities followed by GGO (20%) and consolidation with GGO (20%); this also stated in previous studies that were done by Chen et al. and Liua et al. [8, 15] which reported that consolidation was observed in 50% of the examined children with COVID-19. In contrary to our study, Steinberger et al. [13] reported 0% of consolidation in their studied children with COVID-19. As consolidation accounts for up to half cases in many studies with positive CT findings, it should be considered as a hallmark in diagnosis of pediatric patients with COVID-19 infection.

Early radiology investigative results on COVID-19 in pediatric patients done by Chen et al. and Xia et al. [8, 16] stated that bilateral lung lobes affections were more common than unilateral affection and this is not concomitant with our results, which show that 66.7% of the examined cases with positive CT findings had unilateral lung affection, this can be explained by small number of cases with lung opacities (17%) in our study due to the selection of patients with upper respiratory tract symptoms only. However, the systematic review analysis that was done by Susan et al. [17] reported that unilateral lung affection was more common than bilateral affection as stated in our study. Peripheral distribution of the lung opacities was more frequent than central distribution in our patients that is also greatly consistent with the previous studies [8, 10]. Predominance of lower lung lobes affection were noted in 66.7% of our examined patients that it is consentient with previous studies that were done by Susan et al. [17] and Han et al. [18], as they stated that more than 86% of the pediatric patients with COVID-19 had lower lobe involvement. This could be explained by the anatomy of the lower lung bronchus, which is short in length and thick, making the lower bronchus easy to be catched by the virus.

Recent chest CT report on COVID-19 [8] stated that bronchial wall thickening was more obvious on pediatric patients, and this was also prevalent in our pediatric group (43.4%) and this could be explained by the distribution of the coronavirus infection along the respiratory epithelium in children.

This study has several limitations. Firstly, the sample size was small which limited the study power. Secondly, absence of adult patients group to compare their chest CT features with the pediatric group. Thirdly, the involved patients had only upper respiratory tract infection, so the lung affection was limited.

## Conclusions

RT-PCR is a gold slandered test in the diagnosis of COVID-19 in children with upper respiratory tract symptoms as chest CT cannot standalone as a diagnostic tool owing to high prevalence of normal chest CT in these patients. But, according to our study, we suggest that in case of lung affection, the presence of unilateral, peripheral consolidation with lower lobe predominance, in addition to bronchial wall thickening should be considered as signs of chest CT in pediatric patients with COVID-19 diagnosis.

## Data Availability

The datasets used and/or analyzed during the current study are available from the corresponding author on reasonable request.

## Abbreviations

WHO: World Health Organization
SARS: Severe acute respiratory syndrome
MERS: Middle East respiratory syndrome
CT: Computed tomography
MDCT: Multidetectors computed tomography
RT-PCR: Real-time reverse-transcription–polymerase chain reaction
GGO: Ground-glass opacity
